# Responding to stakeholder needs to engage rehabilitation professionals in the delivery of evidence-based health programming for adults with osteoarthritis

**DOI:** 10.3389/fresc.2022.907477

**Published:** 2022-08-02

**Authors:** Julia Chevan, Maureen Barrett, Kimberly Nowakowski, Kathleen Pappas, Heather Murphy, Elizabeth Erck, Serena Weisner

**Affiliations:** ^1^Department of Physical Therapy, Springfield College, Springfield, MA, United States; ^2^National Association of Chronic Disease Directors, Decatur, GA, United States; ^3^Osteoarthritis Action Alliance, Thurston Arthritis Research Center, The University of North Carolina at Chapel Hill, Chapel Hill, NC, United States

**Keywords:** osteoarthritis, physical activity, self-directed exercise, health coaching, program delivery

## Abstract

Although there are many evidence-based programs that promote healthy lifestyles and symptom modification for people with osteoarthritis, their delivery in rehabilitation clinical settings in the United States is limited. These programs can be a primary component of treatment or a discharge option to facilitate long-term mobility and pain management. The purpose of this perspective article is to describe a delivery model that brings one arthritis-appropriate, evidence-based intervention, the Arthritis Foundation's Walk With Ease program, to older adults seeking physical therapy related to their osteoarthritis. We embedded program delivery into a Doctor of Physical Therapy curriculum using a student health coaching approach and partnering with physical therapy clinics and other community agencies for participant referrals. This model of delivery is cost-effective, sustainable, and provides outcomes that meet goals of the national agenda for osteoarthritis. The model provides benefits for students in health professions education programs, community organizations and rehabilitation clinics, and adults living with osteoarthritis.

## Introduction

Osteoarthritis (OA) is a leading cause of pain and disability among adults in the United States (1) and a condition that is commonly encountered among patients in rehabilitation settings. In outpatient physical therapy settings, 47% of the patient visits are for individuals whose clinical profile includes a diagnosis of OA (2). For these individuals, there is a body of evidence supporting physical activity or a program of exercise and walking as important in symptom management and reduction of disability (3–5). Rehabilitation specialists such as physical and occupational therapists are appropriate professionals to engage in both prescribing and guiding ongoing physical activity and walking programs (6, 7) but there are barriers that prevent these rehabilitation professionals from ongoing therapeutic and health promotion relationships with adults with OA. These barriers include low rates of referral for therapy (8), misperceptions about the role of physical activity in managing OA among physicians and the public (9) and limited or no reimbursement for long-term symptom management, prevention, health promotion, and wellness activities from a rehabilitation specialist (10).

The United States Centers for Disease Control and Prevention (CDC) maintains a menu of arthritis-appropriate evidence-based interventions (AAEBI) which include both physical activity and self-management programs. One of these programs which is supported by clinical research for promoting health and improving function among adults with OA is the Arthritis Foundation's Walk With Ease (WWE) program. WWE is a 6-week walking program developed in the United States by the Thurston Arthritis Research Center and the Institute on Aging of the University of North Carolina (3, 4). The program is typically done in either a group or independently in a structured self-directed format. Studies using WWE as an intervention have shown benefits to participants that include more confidence (11) increased physical activity levels (12), and high levels of satisfaction with the program (13). Callahan (3) compared the outcomes between participants in the two formats (group and self-directed) and found that after 6 weeks all participants had lower scores on measures of disability and pain and higher scores on measures of self-efficacy. In the 1-year follow-up measures the participants in the self-directed format were more likely to continue their walking program and to have better function and symptom management scores (3).

Embedding WWE or other AAEBI into clinical practice for rehabilitation professionals is a desired extension of the evidence. The National Association of Chronic Disease Directors (NACDD) in partnership with the American Physical Therapy Association and the CDC developed several tools and resources (14) for clinicians which provide information on evidence-based programs like WWE. Still, according to staff at the NACDD the uptake by clinics was quite low. In 2017 NACDD provided grant funds to American Physical Therapy Association state chapters in Iowa, Illinois, and Oregon to use clinical facilities and staff to deliver, WWE to people with osteoarthritis. The success in Illinois (15) where the state chapter acted as a delivery hub to foster sustainability and long-term uptake of WWE in physical therapy clinics was expanded in the next iteration of NACDD projects. In the Illinois experience, the hub approach resulted in 22 partner clinics that reached over 5,000 adults (15). In 2019, the NACDD funding call was altered to call for the development of hubs or network centers to disseminate WWE. Springfield College Department of Physical Therapy responded to this call and identified a project goal of integrating WWE into its Doctor of Physical Therapy curriculum and then disseminating that process to other health professions programs. As a theoretical framework to guide our grant efforts we relied on the process theories approach outlined by Grol and Wensing (16, 17). These authors emphasize “a systematic approach and careful planning” with regular evaluation of progress and adaptation to results and challenges consistent with planned-change or planned-action theory. This theoretical approach provided the guidance needed to use the data from external national needs assessments to promote the change in behavior among older adults with OA and to promote learning and change among the health professional students who acted as coaches for the program.

The purpose of this paper is to describe how, through the NACDD grant funded project, Springfield College Department of Physical Therapy met four stakeholder groups' needs with an end goal of promoting physical activity, health and quality of life for adults with OA. We also describe how we managed change in an educational and clinical setting through the regular promotion of an AAEBI as a post episode of care intervention for individuals who sought physical therapy. The stakeholder groups we worked with included organizations at the national level that oversee OA policy and public health outcomes, rehabilitation professional education programs and their students, community organizations and rehabilitation clinics, and individuals who live with OA. Our aim with this descriptive perspective paper is to encourage other health professional training programs (physical therapy, occupational therapy, therapeutic recreation) to develop similar projects that can meet the needs of multiple communities.

## Stakeholders and process

### National organizations that oversee OA policy and public health outcomes in the United States

OA policy and public health outcomes are coordinated by the CDC Arthritis Program (https://www.cdc.gov/arthritis/about/reach.htm) which provides leadership at the national level around the goal of improving the health and quality of life of Americans living with arthritis. The CDC coordinates a national needs assessment for clinical, social, and other services for adults living with arthritis. In 2021, the CDC provided funding to a number of organizations who shared in the goal of improving the lives of Americans living with Arthritis. Three of these stakeholders were the Arthritis Foundation (AF), the NACDD and the Osteoarthritis Action Alliance. Together, the CDC and the three additional stakeholders formed a partnership of collaborating and communicating entities. Each entity addresses distinct but related objectives in meeting public health outcomes for OA as outlined on Table 1.

**Table 1:** Stakeholders funded through CDC initiative to advance arthritis public health priorities.

**Organization**	**CDC funding objectives**
National Association of Chronic Disease Directors (NACDD)	Support innovative efforts that enhance healthcare provider awareness, knowledge, and skills in promoting physical activity as an effective, drug-free way to relieve arthritis pain, improve function, and limit arthritis progression among US adults with arthritis. Training and technical assistance to enhance the capacity of states to effectively address arthritis. Identify best practices and develop tools, resources, and trainings.
Osteoarthritis Action Alliance (OAAA)	Facilitate partnerships and coordinate activities to address national osteoarthritis public health priorities. Maintain and facilitate an active alliance of organizations committed to addressing priorities identified in the National Public Health Agenda for Osteoarthritis
Arthritis Foundation (AF)	Expand provision of tailored consumer arthritis information and appropriate evidence-based interventions through a national Arthritis Helpline.

The CDC and the AF jointly developed the first blueprint for action through the National Public Health Agenda for Osteoarthritis published first in 2010. In 2020, the Osteoarthritis Action Alliance led efforts to update the agenda with input from all stakeholders. The agenda's purpose statement reads “We envision a nation in which adults with OA are able to live full lives with less pain, stiffness, and disability; greater mobility; and preserved function and independence.” This purpose is met through 9 strategies outlined in the Agenda that engage all stakeholders across the country.

The NACDD whose role is clarified in Table 1 has an Arthritis Project that focuses on two initiatives. The first is providing technical assistance and support to the 13 CDC-funded state arthritis programs. The second is working with partners and business influencers on delivering and disseminating arthritis-appropriate evidence-based interventions (AAEBI) including WWE. Strategies undertaken by NACDD to meet this focus include collaborating with rehabilitation specialists such as physical therapists and with the American Physical Therapy Association to encourage creative approaches that will engage health and rehabilitation clinicians to counsel and refer appropriate patients to AAEBIs.

In 2020, Springfield College Department of Physical Therapy entered into this rich stakeholder environment as the recipient of a grant from NACDD to disseminate WWE across the state of Massachusetts and to develop a process to incorporate WWE into a rehabilitation professions curriculum. Though disrupted for a time by the COVID-19 pandemic, the grant proved to be successful in meeting national OA stakeholders' (CDC, AF, NACDD, Osteoarthritis Action Alliance) needs by addressing the following 3 strategies of the National Public Health Agenda for Osteoarthritis:

Strategy 1: Promote evidence-based, self-management programs and behaviors (i.e., self-management education, physical activity, weight management, injury prevention, and health care engagement or provider visits) as nondrug interventions for adults with symptomatic OA.
Met through our unique delivery model for WWE which promotes self-management for physical activity.
Strategy 2: Promote low-impact, moderate-intensity physical activity for adults with OA that includes aerobic, balance, and muscle-strengthening components.
Met through the delivery of the WWE program.
Strategy 5: Expand systems for referral and delivery of evidence-based interventions for adults with OA.
Our program expanded referral and delivery of WWE throughout the state of Massachusetts and in particular engaged physical therapy clinics to make referrals of adults with OA as a component of their discharge planning.

Though small in its initial national impact, our work met the needs of the stakeholder group of national organizations that oversee OA policy and public health outcomes by training future rehabilitation professionals and mobilizing them through their professional level education to deliver one AAEBI. As we describe the ways in which our other stakeholder groups' needs were met it will become evident that the model we developed is scalable and accessible to rehabilitation professions programs and their students across the country.

### Rehabilitation professions programs and rehabilitation professions students

In 2003 the Institute of Medicine published a list of core competencies for health professional education directed at improving the quality of health care delivery (18). One of the 5 core competencies is the delivery of patient-centered care which has been identified as:

“identify, respect, and care about patients' differences, values, preferences, and expressed needs; relieve pain and suffering; coordinate continuous care; listen to, clearly inform, communicate with, and educate patients; share decision making and management; and continuously advocate disease prevention, wellness, and promotion of healthy lifestyles, including a focus on population health.”

We viewed the process of embedding WWE in our curriculum and engaging students in WWE delivery as one effort to meet this core competency. Through our regular curricular evaluation process, we already identified a need to engage students in provision of wellness and health promotion activities. Prior research on student engagement in health coaching models in relation to health program delivery provided guidance on our approach and our anticipation of positive learning outcomes for our students (19–21).

After analyzing the WWE training and certification process, we decided that we would use the self-directed format of WWE and provide our students with the guidance needed to form teams of health coaches for WWE participants. Using the self-directed format enabled us to reach more participants with each session because we didn't need to manage group set-up at a specific facility or have our students travel across the state to coach the participants. Having our students serve in the role of health coaches as opposed to leaders reduced the training costs to our program to certify each student. To embed the WWE program, we conducted an analysis of our curriculum and identified courses with objectives and content that were compatible with this unique student-led, team health coaching approach. In our case, the courses were part of our integrated clinical or experiential curriculum, these are required courses in the DPT curriculum ensuring that all students would engage in this experience.

Faculty and selected graduate student leaders completed training through the AF to become certified WWE leaders. Faculty then wrote a student health coaching manual that had sample scripts for the coaching sessions and that integrated curricular content on communication skills, population health and motivational interviewing as well as pertinent material from both the participant WWE guidebook content and the leader guidelines. Each student coaching team met with a single community participant weekly over the 6 weeks of the self-directed WWE program. Students completed weekly written reflective journaling assignments with prompts (see Table 2 for sample writing prompts) directed to the curricular content of the coaching manual.

**Table 2:** Prompts for student reflections in the learning management system.

**Week**	**Prompt**
WWE week 0—participant orientation	Review the Callahan article specific to the standardized WWE program. How will you explain the research conducted on the WWE program to your participant? Having interacted with your participant and your team, what are the challenges you face, and what are you most looking forward to?
WWE week 1	After reviewing the WWE Student Coaching manual, the WWE resources/links (toolkit), and the Giuffre and Magnusson articles, please respond to each of the following questions: In Table 2, Giuffre et al., provide examples of population-based practices. Can you provide another example for each area of practice? How is the WWE program a population health practice? Magnusson et al., tie population health to health equity and the creation of health-promoting environments. What activities do you currently engage in to promote health equity and create health-promoting environments? How should the profession of Physical Therapy respond to this call for change?
WWE week 2	Consider your session this week in relation to what was described in the systematic reviews completed by Oliveira et al., and Wolever et al. Respond to the following: Have you ever tried to change a health behavior or help a family member change a health behavior? How would health coaching have been of assistance to you in that process?
WWE week 3	Reflect on your discussions with your client this week about coping techniques and overcoming barriers/obstacles. Based on the Cheung article, in which stage of change is your client currently, and discuss your impression of their self-efficacy. Share with your peers what you implemented from the Ivarsson Motivational Interviewing article to meet your client's needs based on the Transtheoretical Model. How did the dialogue feel to you? Did it produce the desired result/participant response? Following your response, respond to one peer from a different group as well.
WWE week 4	Your participant hit the 1/2-way mark. They completed their midpoint self-test. Based on how they are doing, and considering the Simpson article respond to the following: What factors do you believe are most influential on your client's health behavior? How do you think this impacts their readiness to make health behavior changes? How do your client's health beliefs influence their readiness to change? How did your group address these issues during your session this week? Were you successful? Following your primary post, please also respond to a post from a peer.
WWE week 5	Reflect on the Miller article and what motivational interviewing “is not.” Then consider your review session this week with your participant. While motivational interviewing is not based on the transtheoretical model of behavior change; does awareness of this model help to inform the development of your interview and communication style for each session? Please provide examples.
WWE week 6	Review the Rethorn and Pettitt article. Based on your experience with the WWE program respond to the following: Is health coaching “skilled physical therapy care?” How does health coaching fit into a population health model of physical therapy care?

We collected and analyzed data from the students' written reflections and through a series of pre and post semester course evaluation skill assessment surveys. Two researchers reviewed each of the reflections and coded them for major themes. A third researcher then reread the reflections to evaluate themes selected and to assess for saturation. Two main themes derived from student reflections (*n* = 945 postings) and the narrative survey comments include: communication skills and professional growth. Students reported that being a part of a meaningful experience in the provision of community health and wellness was crucial to their professional growth as future rehabilitation professionals. This student comment from the survey is typical of the statements made about growth and is nice evidence of how this stakeholder group's needs were met through the delivery of WWE.

“I've learned from the older adult we worked with. Hearing her experiences has brought me a new perspective on the joys and the challenges of being active later in life.” “Working with our participant each week and hearing the impact I made on them, encourages me to seek out more opportunities like this, where I can serve my peers and the community with the newfound leadership skills I have acquired.”

Students report that having an understanding of an AAEBI and engaging in delivery is also more likely to lead them to implement this practice in their future work as rehabilitation professionals.

### Rehabilitation clinics and community organizations

Rehabilitation clinics provide therapeutic care for adults with OA to manage episodic flare ups of symptoms. At discharge, therapeutic recommendations for self-care often include physical activity but this is not monitored and follow-up is rare. Physical therapy staff in rehabilitation clinics in the United States are not reimbursed for providing the WWE program and this makes it cost prohibitive to embed it directly in clinic activities. The student-coached WWE program was marketed by email messages and phone calls to local physical therapy clinics and facilities as a discharge referral option for adults with OA who had a recent episode of physical or occupational therapy. Through our outreach efforts we connected with clinics and solidified a web-based referral system for adults with OA to the WWE program. Adults with OA benefit from ongoing physical activity programs and at the end of an episode of physical therapy these programs can be part of discharge planning. Through the student coached WWE program, this discharge option was provided at no cost to the clinic and at no cost to the participant.

A second source of referrals and a part of this stakeholder group that also promotes physical activity are local community organizations, such as aging agencies and senior centers. We marketed to these agencies *via* phone call inquiries, emails, informational flyers and virtual presentations. The partnerships we developed with these organizations met their needs in providing a program offering physical activity for adults with OA. Feedback from the agencies was positive and increased awareness of our program across multiple regional organizations thereby increasing referrals for future WWE sessions.

Our student-coached WWE program met these stakeholders' needs by providing a discharge option for rehabilitation clinics and a physical activity program offering for community organizations. It provided trained WWE leaders and required no financial outlay to the clinics and community groups.

### Adults with OA

The most important stakeholder in this project was certainly the adults with OA who are the beneficiaries of the program and its outcomes. To date, we've completed three 6-week sessions of the student-coached, self-directed WWE program with community participants. These participants were recruited from physical therapy clinics, retirement communities, and senior centers. Participants agreed to be matched to a student coaching team and agreed to do the 6-week self-directed WWE program. Grant money from Springfield College covered the cost of the WWE guidebook for each participant. We provided this program to 62 participants who completed the entire 6-week program. Another 23 participants started but withdrew due to personal reasons, illness or technology problems. Although 27% seems a high withdrawal rate we attribute it in part to delivering the program during the COVID-19 pandemic and to our own need to prescreen participants for their ability to navigate the meetings with their student coaching teams.

**Figure 1:**
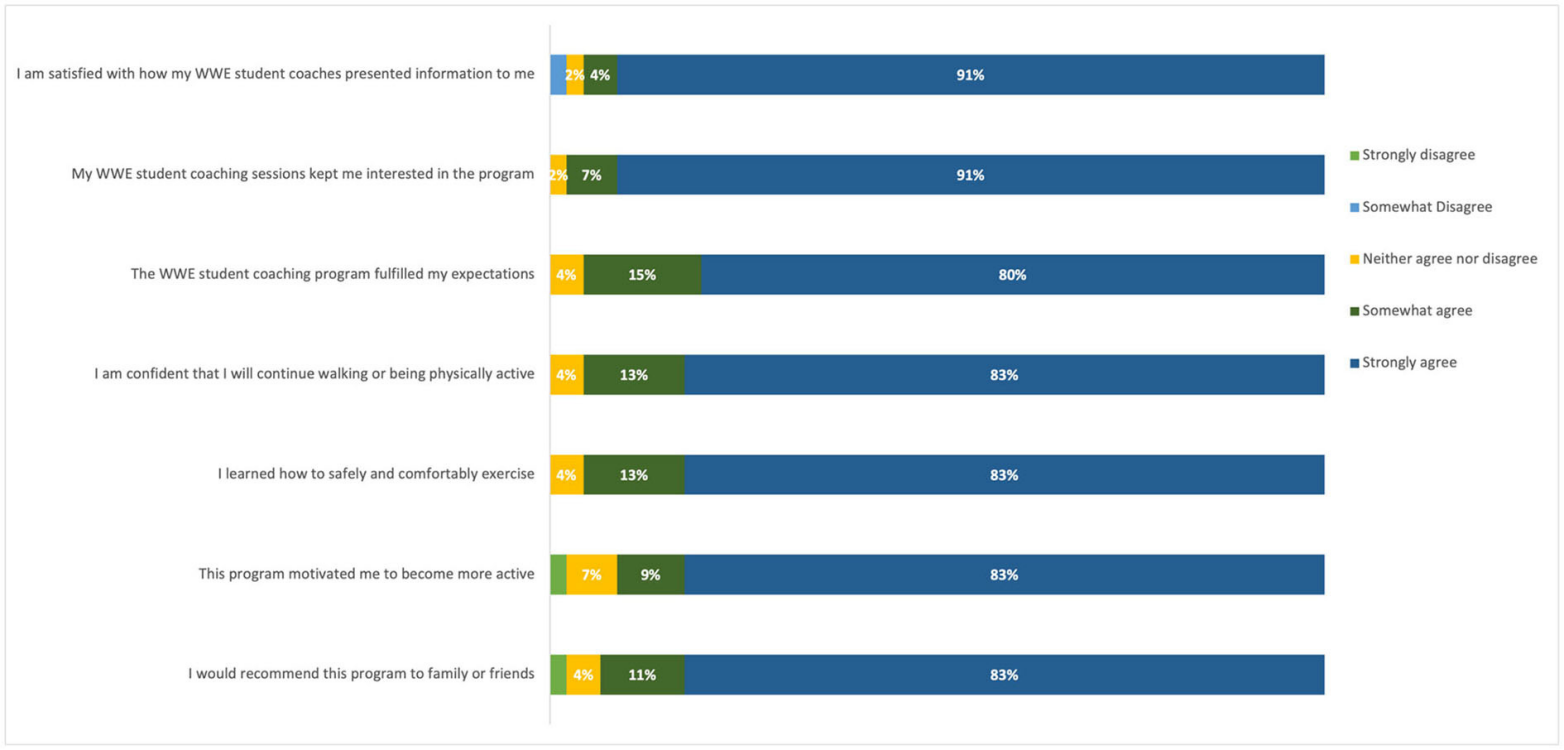
Participant survey results (*N* = 46).

Each of the participants completed a post-session survey and aggregated results can be found on Figure 1. Generally, the evaluative data and comments were positive. The majority of the respondents agreed or strongly agreed that the student-led coaching sessions kept them interested in the program and that they would continue walking or being physically active upon the conclusion of the program. Respondents agreed or strongly agreed that the program itself motivated them to become more active. Positive narrative comments from participants highlighted the student coaches for their roles as motivators and for the accountability they provided. The 3 examples below are typical of these comments.

“Without their coaching and giving me confidence, I wouldn't have been walking,” “I would recommend this program to anyone.”

“This program motivated me to get out and walk when I didn't really feel like doing so.”

The participant as a stakeholder is truly at the center of the AAEBIs and of the WWE program. The student coached model met stakeholder needs for OA symptom management, for improved mobility and provided motivation and accountability as a supplement. Participant stakeholders were satisfied and many have requested a second round in the program.

## Lessons learned

Embedding an AAEBI into a curriculum needs to be done in an intentional manner so that curricular objectives and student learning outcomes are met. Using the planned-change theory to frame the work ensures that adaptations can be made as the program is introduced (16, 17). The costs of embedding the program revolve around faculty time which is focused on organizing the program, recruiting participants, and supervising students. Communication with the rehabilitation professional and the community organizations prior to each planned 6-week WWE session is needed in order to recruit participants. Additional time is needed to reorganize student health coaching teams when there is participant attrition.

Participant knowledge and use of technology can be a limiting factor when virtual delivery of coaching sessions is required as was the case during the COVID-19 pandemic. Students found that some participants did not fully understand the use of technology, requiring additional time to problem solve and instruct the participants during the planned WWE session. Flexibility and adaptability of students to meet participant needs is imperative. Students found that some participants had limited availability such as meeting prior to 8:00 A.M. or only during lunch, or some participants took vacations in the midst of the sessions.

Additional faculty time is required to consult with students to triage unexpected situations or concerns that arise and seem to be outside of the WWE program scope. Students were able to recognize when a participant's question needed to be answered by faculty. Faculty were typically consulted when there was a change in a participant's medical status, a situation that affected their participation in the program, or other questions related to issues outside of the WWE program that participants shared. These consults served as an opportunity for faculty to mentor students in professional communication, both written and verbal, as well as to demonstrate empathy and identify scope of practice—all of which are important within the curriculum, but require time and faculty commitment.

Sustainability of this model of AAEBI program delivery can be accomplished through embedding the delivery permanently into the health profession's curriculum as a mandatory requirement. This requires identification of the courses and objectives that are compatible with a student-led health coaching model. Without grant funding this can occur using department pro bono clinics, through service-learning activities or as a component of clinical experiences.

## Conclusions

AAEBIs such as WWE can be provided as part of a curriculum of a health profession's academic program. Ideally, the program partners with community clinical sites who regularly discharge and refer adults with OA. This engages rehabilitation professionals in ensuring that adults with OA are meeting physical activity requirements. The WWE program efficacy (3, 4, 12, 13) for adults with OA was previously established and thus not a focus of our project. Still, participants reported positive experiences and a commitment to ongoing physical activity. Students involved in this student-led health coaching model also reported positive experiences and understanding of health and wellness programming. Students enter professional practice valuing the importance of AAEBIs and the skills necessary to deliver programs such as WWE within their communities thus contributing to sustainability.

After piloting and then running our program over a 2-year period we felt comfortable sharing the curriculum and the student coaching manual with other academic programs. The student coaching manual is available on our website (https://springfield.edu/walk-with-ease/dpt) and is currently being used by at least 3 academic physical therapy programs in the United States. We believe the program could be adopted by other academic rehabilitation programs including occupational therapy and therapeutic recreation.

## Data availability statement

The original contributions presented in the study are included in the article/supplementary materials, further inquiries can be directed to the corresponding author/s.

## Ethics statement

This study was reviewed and approved by the Springfield College IRB. Written informed consent for participation was not required for this study in accordance with the national legislation and the institutional requirements.

## Author contributions

All authors listed have made a substantial, direct, and intellectual contribution to the work and approved it for publication.

## Funding

This project was supported by the Centers for Disease Control and Prevention of the U.S. Department of Health and Human Services (HHS) as part of a financial assistance award totaling $2,000,000 with 100% funded by CDC/HHS.

## Conflict of interest

The authors declare that the research was conducted in the absence of any commercial or financial relationships that could be construed as a potential conflict of interest.

## Publisher's note

All claims expressed in this article are solely those of the authors and do not necessarily represent those of their affiliated organizations, or those of the publisher, the editors and the reviewers. Any product that may be evaluated in this article, or claim that may be made by its manufacturer, is not guaranteed or endorsed by the publisher.

## Author disclaimer

The contents are those of the author(s) and do not necessarily represent the official views of, nor an endorsement, by CDC/HHS, or the U.S. Government.

## References

[1] United States Bone Joint Initiative. The Burden of Musculoskeletal Diseases in the United States (BMUS). (2020). Available online at: http://www.boneandjointburden.org (accessed February 20, 2022).

[2] Machlin SR Chevan J Yu WW Zodet MW. Determinants of utilization and expenditures for episodes of ambulatory physical therapy among adults. Phys Therapy. (2011) 91:1018–29. 10.2522/ptj.20100343 21566066

[3] Callahan LF Shreffler JH Altpeter M Schoster B Hootman J Houenou LO. Evaluation of group and self-directed formats of the Arthritis Foundation's Walk With Ease program. Arthritis Care Res. (2011) 63:1098–107. 10.1002/acr.20490 21560255

[4] Callahan LF Mielenz T Freburger J Shreffler J Hootman J Brady T. A randomized controlled trial of the people with arthritis can exercise program: Symptoms, function, physical activity, and psychosocial outcomes. Arthritis Rheumatism. (2008) 59:92–101. 10.1002/art.23239 18163409

[5] Nyrop KA Charnock BL Martin KR Lias J Altpeter M Callahan LF. Effect of a six-week walking program on work place activity limitations among adults with arthritis. Arthritis Care Res. (2011) 63:1773–6. 10.1002/acr.20604 22127968

[6] Brakke R Singh J Sullivan W. Physical therapy in persons with osteoarthritis. PM&R. (2012) 4:S53–8. 10.1016/j.pmrj.2012.02.017 22632703

[7] Kolasinski SL Neogi T Hochberg MC Oatis C Guyatt G Block J. 2019 American College of rheumatology/arthritis foundation guideline for the management of osteoarthritis of the hand, hip, and knee. Arthritis Rheumatol. (2020) 72:220–33. 10.1002/art.41142 31908163 PMC10518852

[8] Iversen MD Schwartz TA von Heideken J Callahan LF Golightly YM Goode A. Sociodemographic and clinical correlates of physical therapy utilization in adults with symptomatic knee osteoarthritis. Physical Therapy. (2018) 98:670–8. 10.1093/ptj/pzy052 29718472 PMC6057494

[9] Poitras S Rossignol M Avouac J Avouac B Cedraschi C Nordin M. Management recommendations for knee osteoarthritis: How usable are they? Joint Bone Spine. (2010) 77:458–65. 10.1016/j.jbspin.2010.08.001 20851659

[10] U.S. Centers for Medicare & Medicaid Services. Chapter 15 – Covered medical other health services 220.2 D. In *Medicare Benefit Policy Manual [Internet]. Rev 11288*. Baltimore: U.S. Centers for Medicare & Medicaid Services (2022).

[11] Bruno M Cummins S Gaudiano L Stoos J Blanpied P. Effectiveness of two arthritis foundation programs: walk with ease, and you can break the pain cycle. Clin Intervent Aging. (2006) 1:295–306. 10.2147/ciia.2006.1.3.295 18046884 PMC2695175

[12] Conte KP Odden MC Linton NM Harvey SM. Effectiveness of a scaled-up arthritis self-management program in Oregon: walk with ease. Am J Public Health. (2016) 106:2227–30. 10.2105/AJPH.2016.303478 27736216 PMC5105015

[13] Nyrop KA Cleveland R Callahan LF. Achievement of exercise objectives and satisfaction with the walk with ease program—group and self-directed participants. Am J Health Promotion. (2014) 28:228–30. 10.4278/ajhp.120920-ARB-453 23875982

[14] American Physical Therapy Association,. Arthritis Management Community-Based Programs. (2019). Available online at: https://www.apta.org/patient-care/public-health-population-care/arthritis-management

[15] National Association of Chronic Disease Directors. Walking their way to success: The Illinois Physical Therapy Foundation. (2019). Available online at: https://chronicdisease.org/wp-content/uploads/2021/04/NACDD-Success-Story-Design-FINAL.pdf

[16] Grol R Bosch MC Hulscher MEJL Eccles MP Wensing M. Planning and studying improvement in patient care: the use of theoretical perspectives. Milbank Q. (2007) 85:93–138. 10.1111/j.1468-0009.2007.00478.x 17319808 PMC2690312

[17] Grol R Wensing M Ecclees M Davis D. Improving Patient Care: The Implementation of Change in Health Care. 2nd ed. Oxford: John Wiley & Sons (2013). 10.1002/9781118525975

[18] Greiner AC Knebel E. Institute of Medicine (US) Committee on the Health Professions Education Summit. Washington DC: National Academies Press (2003). 25057657

[19] Krok-Schoen JL Shim R Nagel R Lehman J Myers M Lucey C. Outcomes of a health coaching intervention delivered by medical students for older adults with uncontrolled type 2 diabetes. Gerontol Geriatrics Educ. (2017) 38:257–70. 10.1080/02701960.2015.1018514 25701102 PMC4545471

[20] Phillips EA. Evaluation of a coaching experiential learning project on OT student abilities and perceptions. Open J Occupat Ther. (2017) 5:e1256. 10.15453/2168-6408.1256

[21] Ickes MJ McMullen J. Evaluation of a health coaching experiential learning collaboration with future health promotion professionals. Pedagogy Health Promotion. (2016) 2:161–9. 10.1177/2373379916649193

